# Reviewing physical exercise in non-obese diabetic Goto-Kakizaki rats

**DOI:** 10.1590/1414-431X2022e11795

**Published:** 2022-05-27

**Authors:** B.S.M. Galán, T.D.A. Serdan, L.E. Rodrigues, R. Manoel, R. Gorjão, L.N. Masi, T.C. Pithon-Curi, R. Curi, S.M. Hirabara

**Affiliations:** 1 Programa de Pós-Graduação Interdisciplinar em Ciências da Saúde, Instituto de Atividade Física e Esportes, Universidade Cruzeiro do Sul, São Paulo, SP, Brasil; 2 Department of Molecular Pathobiology, New York University, New York, NY, USA; 3 Seção de Produção de Imunobiológicos, Centro Bioindustrial, Instituto Butantan, São Paulo, SP, Brasil

**Keywords:** Exercise intensity, Insulin resistance, Glycemic control, Type 2 diabetes mellitus, Skeletal muscle, Metabolism

## Abstract

There is a high incidence of non-obese type 2 diabetes mellitus (non-obese-T2DM) cases, particularly in Asian countries, for which the pathogenesis remains mainly unclear. Interestingly, Goto-Kakizaki (GK) rats spontaneously develop insulin resistance (IR) and non-obese-T2DM, making them a lean diabetes model. Physical exercise is a non-pharmacological therapeutic approach to reduce adipose tissue mass, improving peripheral IR, glycemic control, and quality of life in obese animals or humans with T2DM. In this narrative review, we selected and analyzed the published literature on the effects of physical exercise on the metabolic features associated with non-obese-T2DM. Only randomized controlled trials with regular physical exercise training, freely executed physical activity, or skeletal muscle stimulation protocols in GK rats published after 2008 were included. The results indicated that exercise reduces plasma insulin levels, increases skeletal muscle glycogen content, improves exercise tolerance, protects renal and myocardial function, and enhances blood oxygen flow in GK rats.

## Introduction

### Type 2 diabetes mellitus: lean *vs* obese patients

Excessive accumulation of adipose tissue has been considered a public health problem for the last 50 years and today has reached epidemic proportions globally. This condition results from a positive energy balance, in which energy intake exceeds energy expenditure. Indeed, it has been shown that excessive food intake and insufficient physical activity reduce the ability of peripheral tissues to respond to insulin, a process known as insulin resistance (IR) (1).

Obesity is a multifactorial disease associated with various genetic and environmental factors. Obese individuals have an increased risk of developing chronic disorders such as hypertension, cardiovascular disease, IR, metabolic syndrome, and type 2 diabetes mellitus (T2DM). Notably, individuals with obesity-related diseases display more severe symptoms and suffer from higher mortality rates when infected with viruses such as COVID-19 (2).

It has been known for decades that obesity leads to T2DM. This disease is a public health problem characterized by high blood sugar concentrations (hyperglycemia) associated with impaired insulin sensitivity in the liver, skeletal muscle, and adipose tissue and reduced insulin secretion (3,4). It is estimated that T2DM corresponds to 90-95% of all diabetes cases (5). Furthermore, obese T2DM patients typically present a low-grade and chronic inflammation (6,7), including pro-inflammatory cytokines (TNF-α, IL-6, IL-8, and IFN-α) and C-reactive protein (CRP).

For example, several lines of evidence indicate that adipsin is a fat inflammation-related and visceral fat accumulation biomarker (8,9). Other biomarkers, including plasma leptin, resistin, tumor necrosis factor-alpha (TNF-α), plasminogen activator inhibitor 1(PAI-1), interleukins (IL) IL-1β, IL-6, and IL-8, insulin-like growth factor 1 (IGF-1), monocyte chemoattractant protein 1 (MCP-1), and visfatin, have also been reported. The plasma IL-6 concentration was proposed to be a T2DM and cardiovascular disease marker associated with an inflammatory state, and increased systemic IL-6 concentrations have been correlated with increased fat mass in both rodent models and obese humans (4,10) . Moreover, reduced serum adiponectin levels in obese individuals were associated with T2DM and attenuated cardiovascular function (11).

It is well known that chronic inflammation reduces the peripheral insulin response, promoting resistance to the hormone, and, in TNF-α knockout rats, improved insulin sensitivity has been reported. Additionally, obese TNF-α knockout mice have reduced fat pad weights and enhanced responses to exogenous glucose and insulin than obese mice with unaltered peripheral insulin sensitivity. These results are due to high tissue TNF-α levels promoting insulin receptor substrate-1 (IRS-1) serine phosphorylation rather than the tyrosine kinase-dependent phosphorylation, ultimately inhibiting insulin signaling (12).

In the last 25 years, the prevalence of obese T2DM has increased from 13 to 31% in eastern populations (5,13). However, T2DM also occurs in non-obese individuals (14). For example, in Asia, 70% of T2DM patients are non-obese with body mass index (BMI) values of <19 kg/m^2^ (15,16) and present the ketosis-resistant diabetes young (KRDY) phenotype, which is associated with a high propensity for beta-cell function failure. Notably, KRDY patients have a higher mortality rate compared to obese subjects.

A similar disease feature was reported in a comparative study of lean and obese diabetic patients in the United States (17). The lean patients from the US study exhibited an increased propensity for pancreatic beta-cell failure, a low total triglycerides/high-density lipoprotein ratio (TG/HDL) (an indirect marker of hepatic IR), and central obesity (i.e., hip circumference) (17). Moreover, in a study with non-obese Filipino T2DM patients, Bautista et al. (18) detected reduced pancreatic beta-cell function using the Homeostasis Model Assessment (HOMA) index compared to overweight/obese patients. In non-obese diabetic patients, the impaired pancreatic beta-cell function is associated with genetic factors, including different alleles in Japanese and Chinese populations (19,20), and acquired autoimmunity, requiring exogenous insulin administration for glycemic control (17) . Since environmental and genetic factors are associated with non-obese T2DM development, future studies will need to be conducted to understand the underlying pathophysiological mechanisms associated with this disease.

### Physical exercise in T2DM subjects

Sedentarism is one of the main factors for developing metabolic syndrome, cardiovascular diseases, and T2DM. Studies have shown that regular physical activity reduces the prevalence and helps manage these obesity-related diseases (21,22). For example, regular physical activity improved glucose tolerance, blood lipid level profiles, and reduced risk factors for developing cardiovascular diseases in T2DM patients (23). Additionally, physical exercise programs have been shown to ameliorate glucose uptake via insulin-independent translocation and glucose transporter 4 (GLUT-4) expression, potentiate insulin signaling during the post-exercise period, increase skeletal muscle oxidative capacity, attenuate lipid metabolite generation, up-regulate the expression of genes associated with mitochondrial biogenesis and metabolism, decrease inflammatory markers, and restore immune function (24-27). Interestingly, improved mitochondrial function and biogenesis have also been observed in the skeletal muscle of diabetic rats and humans following exercise training (28,29).

Furthermore, physical exercise reduces cell hypertrophy and hyperplasia in adipose tissue and increases GLUT-4 expression and insulin sensitivity (30). It has also been shown to increase IL-6, IL-1ra, and IL-10 and decrease TNF-α plasma levels, with an inflammation modulatory effect (31,32). Additionally, the activation of signaling pathways due to muscle contraction increases glucose and fatty acid (FA) metabolism through gene expression regulation. For example, during physical exercise, AMP-activated protein kinase (AMPK) and calcium-calmodulin-dependent protein kinase (CaMK) are activated (33).

Exercise-induced AMPK activation has been shown to lead to 1) reductions in malonyl-CoA generation, which inhibits carnitine palmitoyltransferase 1 (CPT-1), by phosphorylating and inhibiting acetyl-CoA carboxylase, consequently increasing FA oxidation acutely; 2) increased insulin-independent glucose uptake due to GLUT-4 translocation to the plasma membrane; and 3) up-regulated expression of genes related to mitochondrial metabolism and biogenesis, resulting in persistently increased FA oxidation (34). CaMK regulates genes associated with mitochondrial biogenesis induced by muscle contraction (33,35).

### GK rats: a lean model of type 2 diabetes mellitus

The Goto-Kakizaki (GK) rat, a spontaneous non-obese-T2DM animal model, was generated by selective inbreeding using glucose-intolerant Wistar rats with a hyperglycemic phenotype (15). Many studies have utilized GK rats to evaluate non-obese T2DM features, development, and complications (3,4,36-39). These rats display insulin sensitivity impairment and T2DM similar to high-caloric diet-induced obese animals. Interestingly, in contrast to the changes in rats submitted to diet-induced obesity, the increased expression of proinflammatory cytokines is not observed in the skeletal muscle and retroperitoneal adipose tissue depots of 16-week-old GK rats (3,38).

GK rats develop T2DM earlier in life, exhibiting a reduced pancreatic beta-cell mass at 16.5 days of fetal age. At 28 days of age, basal hyperglycemia, IR, and impaired insulin secretion by pancreatic beta-cells are observed. However, GK rats present a preserved beta-cell response to other secretagogues, increased hepatic glucose production, liver inflammation and liver glucotoxicity, and late-stage diabetes complications (3,15,37,40).

GK rats also exhibit typical T2DM hallmarks such as hyperinsulinemia, increased gluconeogenesis, and elevated plasma lipid levels (3,36). Indeed, impaired skeletal muscle oxidative capacity has been associated with T2DM pathogenesis in non-obese individuals. It has been suggested that the lower skeletal muscle oxidative capacity and attenuated mRNA expression of the nuclear receptor coactivator PGC1-α is related to impaired mitochondrial function (41,42).

Xue et al. (43) studied GK rats from 4-20 weeks of age and reported that 412 genes were differentially expressed in white adipose tissue (WAT). This result is not entirely surprising, given that a progressive failure of body fat accumulation was associated with animal age. The authors also detected down-regulated gene expression of fatty acid synthase (Fasn) and signal transducer and activator of transcription-3 (Stat-3), a transcription factor activated by leptin, and up-regulated expression of inflammatory response and interferon-regulated genes. Overall, the GK rats exhibit increased caloric intake, less body fat accumulation, and chronic inflammation in the absence of obesity.

In pancreatic beta-cells, ATP-sensitive K^+^ (K_ATP_) channel closure, due to plasma membrane depolarization and voltage-dependent calcium channel opening, is crucial for glucose-stimulated insulin secretion (GSIS) (44,45). Alterations in K_ATP_ channel activity can lead to changes in plasma membrane polarization, impaired insulin secretion, and diabetes mellitus. In GK rats, insufficient K_ATP_ channel closure caused by reduced glucose metabolism in pancreatic beta-cells culminates in GSIS impairment (46).

Our group recently described marked changes in the three small intestine segments (duodenum, jejunum, and ileum) of four-month-old male GK rats (39). Compared to Wistar rats, GK rats have a reduced small intestine, diminished crypt depth in the duodenum and ileum, increased crypt depth in the jejunum, and longer and thicker villi in the jejunum and ileum. The three intestine segments also had thicker muscular layers. At the molecular level, GK rats display elevated IL-1β levels in the duodenum and jejunum and elevated NF-κB p65 in all three segments. These changes are associated with a high IL-1β reactivity in the muscle layer, myenteric neurons, and glial cells. Moreover, a significant reduction in submucosal neuron density in the jejunum and ileum, ganglionic hypertrophy in all three intestinal segments, and a slower intestinal transit were observed. It was concluded that IR and T2DM development in GK rats is associated with small intestine morphology changes, tissue inflammation, and decreased intestinal transit.

Considering the evidence discussed above, along with the fact that regular physical activity combined with weight loss improves glucose homeostasis by raising glucose disposal and insulin action in obese T2DM patients (47), this review sought to evaluate the literature reporting the effects of physical exercise on diabetic features of GK rats.

## Methodology

### Physical activity/exercise practice in GK rats

#### Eligibility criteria and key points for selecting the related studies

The PICO (Patient, Intervention, Comparison and Outcome) strategy was used to develop and write this narrative review. This approach reduces the possible risks of bias in the article selection process. This study reviewed and analyzed the published literature on chronic physical exercise interventions in GK rats, focusing on metabolic and physiological alterations. The study results are relevant to understanding how physical exercise can specifically benefit non-obese T2DM patients.

Two review team researchers independently searched electronic databases [PubMed, MedLine, Cochrane, Web of Science, and Scopus] from January to November of 2021 for studies employing T2DM GK rats of any age OR sex AND involving voluntary OR non-voluntary exercise training OR other types of physical activity OR exercise OR exercise training AND non-obese type 2 diabetes mellitus *OR* muscle electrostimulation from 2008 to 2021. We limited the search to include randomized controlled trials published only in English in international indexed journals.

The articles used in this review were selected by two independent researchers and based on the titles, objectives, methodologies, and results of each article. Discrepancies between the two researchers were resolved by discussion with a third researcher. At the end of the selection process, 16 studies were identified as being associated with the evaluation of the effects of physical activity or exercise training intervention on non-obese T2DM GK rats.

**Figure 1:**
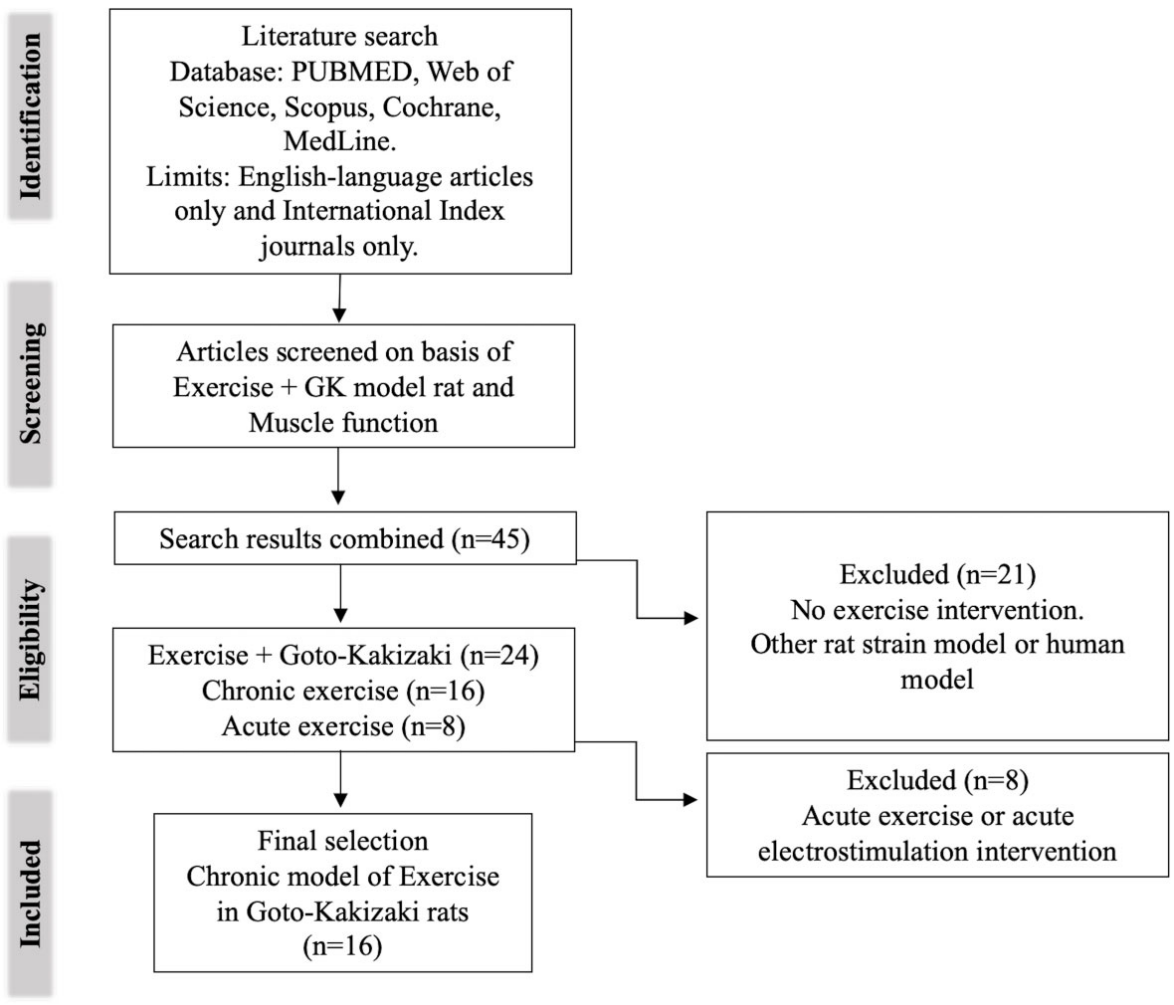
Flow chart of the study selection process for review preparation. GK: Goto-Kakizaki.

**Figure 2:**
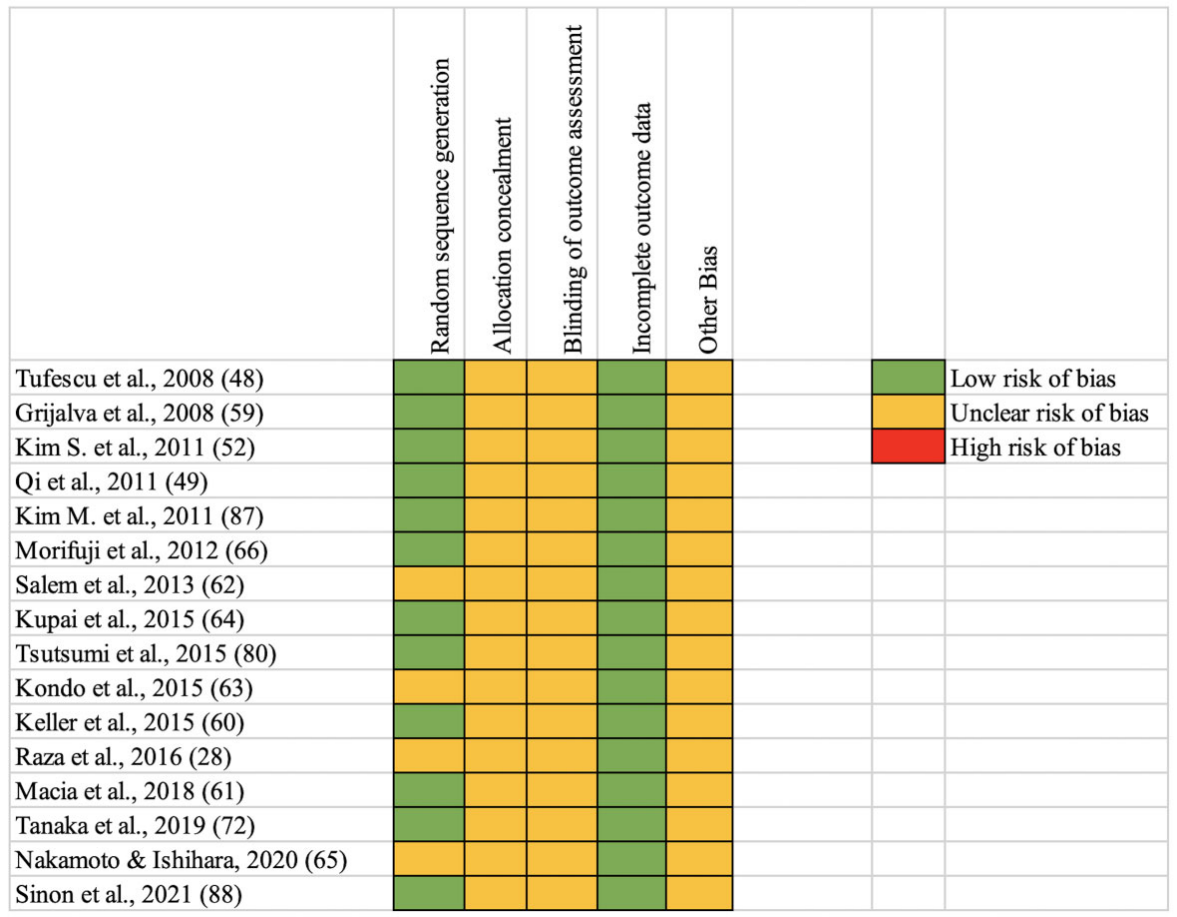
Risk of bias assessments for review studies of exercise intervention in Goto-Kakizaki rats. Low risk of bias: if present, is unlikely to alter the results seriously; unclear risk of bias: a risk of bias that raises some doubt about results; high risk of bias: bias may alter the results seriously.

A flow chart of the search strategy is presented in Figure 1, and a summary of the risk of bias is presented in Figure 2. The topics in this review, including obesity, insulin resistance, physical exercise, type 2 diabetes, and metabolism, are supported by solid research and vast literature. Thus, the selected articles facilitate the discussion of the central theme.

### Main effects of physical exercise in GK rats

#### Effects on metabolic and diabetic features

As shown in Supplementary Table S1, the exercise training protocols included moderate-intensity swimming (n=1), moderate-intensity treadmill running (n=9), low-intensity treadmill running (n=3), low-intensity voluntary wheel activity (n=2), and blood restriction and muscle stimulation (n=1).

In general, moderate physical activity markedly improved the diabetic features of GK rats. Tufescu et al. (48) reported that treadmill exercise reduced urinary protein excretion, indicating renoprotective effects, and enhanced type I fiber capillarization and proportion in extensor digitorum longus (EDL) muscle. Type I fibers exhibit a high capacity for oxidative metabolism and are resistant to fatigue. Qi et al. (49) reported that 10-12 week-old GK rats, submitted to 30-60 min of moderate-intensity treadmill exercise six days per week for eight weeks, displayed no differences in body mass or blood glucose levels but had increased serum adiponectin and insulin levels. Moreover, in skeletal muscle, the authors observed enhanced cytochrome *c* oxidase (COX) activity and mitochondrial DNA (mtDNA) markers, such as COXII protein content, and attenuated p53 protein and tumor p53-induced glycolysis and apoptosis regulator (TIGAR) expression levels.

It is well known that p53 protein regulates aerobic metabolism and is involved in various steps of glycolysis, inhibiting glucose transport through down-regulated GLUT-1 and GLUT-4 gene expression (49,50). Additionally, p53 is involved in mitochondrial function, mtDNA content, and biogenesis. The lack of this protein has been associated with low oxygen consumption and high lactate production, favoring substrate flux through the glycolytic pathway to maintain the required ATP production (51).

In skeletal muscle of 46-52-week-old GK rats, treadmill training increased the protein levels of phosphorylated AMPK, peroxisome proliferator-activated receptor coactivator 1 alpha (PGC-1α), GLUT-4, lactate dehydrogenase (LDH), monocarboxylate transporters 1-4 (MCT-1 and -4), and COX-IV (52). The same study also reported increased glycogen content and citrate synthase activity in the liver and skeletal muscle of rats subjected to exercise training (52). These results corroborated reports that exercise-induced glucose uptake (53) correlates with skeletal muscle LDH and MCT-1 activities, oxidative capacity, and lactate oxidation. It has been reported that the up-regulation of LDH, MCT-1, and COX-IV protein levels reduces hyperlactatemia in diabetic animals after an exercise session. PGC-1α is involved in mitochondrial biogenesis, insulin sensitivity, and lipid metabolism, playing a central regulator of phenotypic adaptation and substrate utilization induced by physical exercise (54). AMPK is involved in the activation of mitophagy, autophagy, and lipolysis. AMPK also inhibits other metabolic pathways, such as lipogenesis, protein synthesis, and gluconeogenesis (55,56). Notably, Kim et al. (52) reported improved glucose homeostasis, oxidative metabolism, IR, and up-regulated AMPK, PGC-1α, and GLUT-4 protein expression in GK rats after exercise training.

Nitric oxide synthase (NOS) produces nitric oxide (NO), which plays a crucial role in nervous system function and vascular tone (57). Increased blood glucose levels are associated with impaired NO production and altered endothelial NOS (eNOS) and nicotinamide adenine nucleotide phosphate (NADPH) oxidase activities (58). Indeed, it has been demonstrated that humans and animals with T2DM present impaired mitochondrial oxidative capacity (58). Thus, it is plausible that the benefits of moderate exercise on the mitochondrial oxidative capacity of T2DM subjects are mediated by increased NO production (59). Interestingly, a study performed with plantaris muscle and left cardiac ventricle of 9-week-old GK rats submitted to 9 weeks of running exercise reported enhanced insulin sensitivity and eNOS expression (59). The same authors showed improved mitochondrial biogenesis and function through increased eNOS, PCG1-α, and UCP3 (uncoupling protein 3) protein expression in the thoracic aorta of GK rats subjected to moderate-intensity exercise (59).

Another study performed with 18-week-old T2DM rats submitted to 8 and 14 days of physical exercise with or without saxagliptin, an inhibitor of glucagon-like peptide 1 (GLP-1) degradation, investigated eNOS activity stimulation (60). The authors found that exercise training improved mitochondrial function and markers in older T2DM rats. Raza et al. (28) reported that eight weeks of moderate-intensity exercise inhibited NADPH oxidase activity, reactive oxygen species (ROS) production, and superoxide dismutase (SOD) activity in the pancreas of 11-month-old GK rats. These results suggested improved mitochondrial function due to enhanced oxygen utilization via the modulation of mitochondrial complex IV activity and PPAR-γ levels.

Seven-month-old T2DM rats previously submitted to 8 weeks of treadmill running presented improved insulin sensitivity and citrate synthase activity in the gastrocnemius muscle (61). Additionally, the ventricular myocyte of 11-month-old GK rats submitted to 2-3 months of treadmill exercise exhibited increased intercellular Gap junctional (Gja1) expression, a gene involved in heart contraction. It should be pointed out that the increased Gja1 expression was not followed by altered calcium (Ca^2+^) transport (62), indicating that exercise intervention preserves the ventricular shortening of myocytes and Ca^2+^ transport (62).

Several authors described the beneficial effect of physical exercise intensity in obese-associated T2DM rat models (61,62). However, only three studies evaluated the efficacy of low-intensity exercise in GK rats (63-65). Kupai et al. (64) observed cardio-protective effects in GK rats after six weeks of voluntary exercise, which could be associated with increased NOS activity in cardiac and aortic tissues. After three weeks of treadmill running, Kondo et al. (63) observed a PGC-1α-induced increase in mitochondrial function in GK rat muscle. Moreover, after six weeks of treadmill running, GK rats exhibited enhanced mitochondrial oxidative capacity due to the increased succinate dehydrogenase (SHD) activity, an enzyme associated with microangiopathy prevention (66). Nakamoto et al. (65) also reported increased SHD activity and detected up-regulated PGC1-α expression in the soleus muscle of six-week-old GK rats after six weeks of voluntary wheel running. Exercise training increased this animal model's oxidative activity and oxygen uptake in skeletal and cardiac muscles (67). These results are consistent with these tissues exhibiting high energy metabolism levels (67,68) and suggest that low-intensity exercise enhances mitochondrial function and oxidative activity in skeletal and cardiac muscle of lean T2DM subjects.

**Figure 3:**
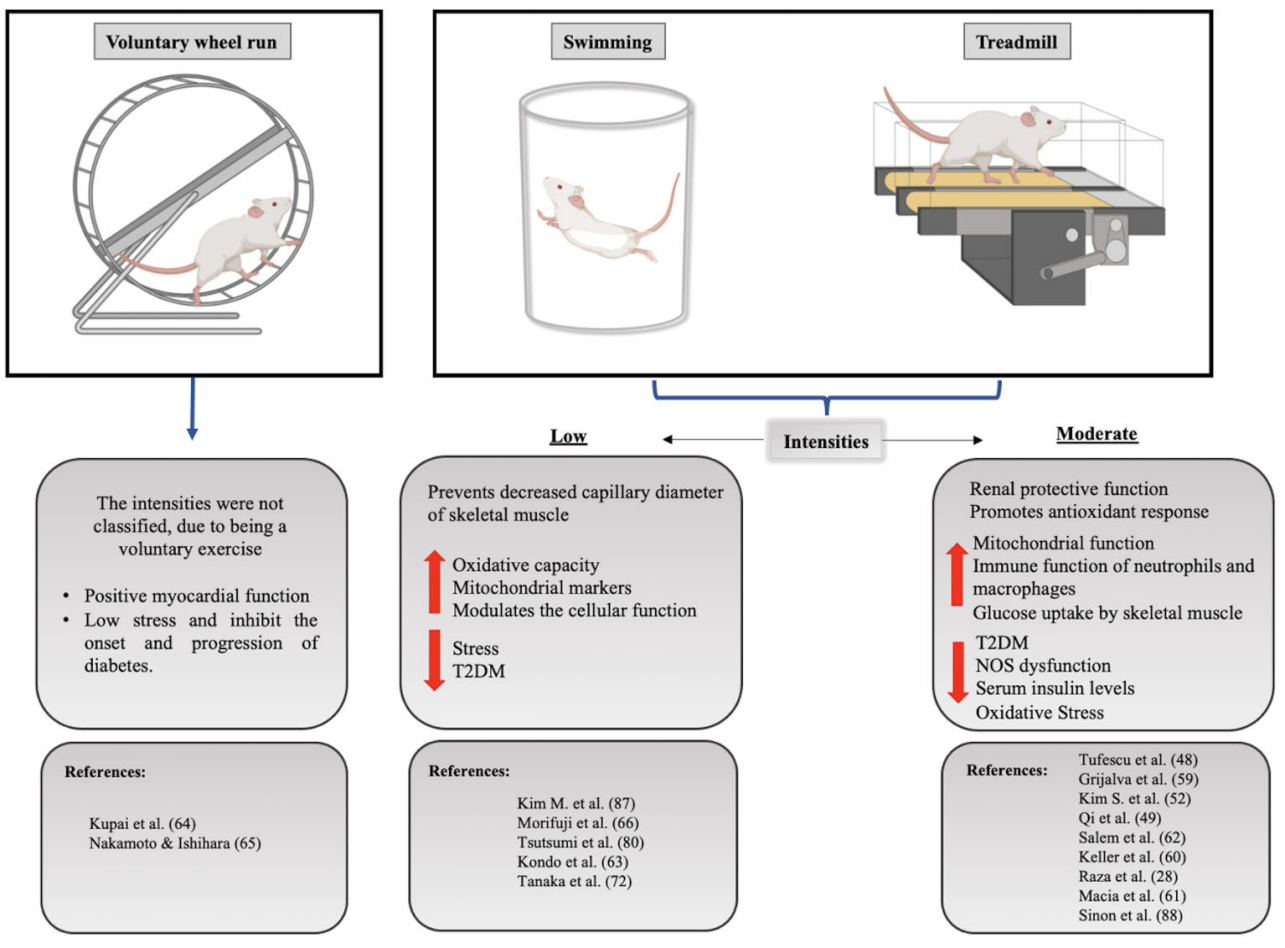
Main findings reported in the reviewed articles on physical exercise in Goto-Kakizaki (GK) rats.

Previous studies have also shown that hyperglycemia generates advanced glycation end products (AGEs), which have been shown to accumulate in tissues (69) and are associated with the down-regulation of skeletal muscle protein synthesis and subsequent skeletal muscle atrophy (70). Using diabetic subjects, Goldin et al. (71) demonstrated that AGEs inhibit NO activity, increase ROS production, and impair vasculature. A study performed with GK rats submitted to a combined electro-stimulation treatment and blood flow restriction reported attenuated AGE production and skeletal muscle protein synthesis activation (72). Based on these findings, it appears that even low-intensity physical exercise reduces AGE levels and could prevent skeletal muscle atrophy in lean T2DM individuals. The exercise training protocols reported in the reviewed articles are summarized in Figure 3.

#### Varying beneficial effects according to exercise intensity in GK rats

Exercise intensity refers to the amount of energy required for the performance of physical activity per unit of time. Exercise intensity can be measured directly using respiratory gas analysis to quantify oxygen uptake during exercise or through standard regression models to estimate energy expenditure per a given work rate. Another way to express exercise intensity is as multiples of resting oxygen requirement [metabolic equivalents (METs)]. In this approach, one MET corresponds to the amount of oxygen consumed by a resting, awake individual and is equivalent to 3.5 mL O_2_ per kg body weight per minute. According to previous studies, low-intensity exercise corresponds to activities requiring <3 METs, moderate-intensity requires 3-6 METs, and high-intensity requires >6 METs (73). The American College of Sports Medicine describes moderate-intensity exercise ranges between 40 and 60% of the maximal capacity, whereas high-intensity exercise is above 64% of the maximal capacity. Most studies describe high-intensity exercise as above 80-85% of peak power output or maximal velocity. In general, activities requiring <80% of peak output are not considered high-intensity training (74,75).

However, in rats, the exercise intensity tolerance varies depending on the rat strain and associated disease, reflecting a different work intensity and probably altering the final metabolic and physiologic assessments if the correct intensity for each rat strain and comorbidity is not utilized. It is also necessary to consider other variables, such as treadmill inclination, speed, oxygen consumption (VO_2_), and/or intensity tracker. Despite these potential challenges, all protocols included in this review demonstrate that low- to moderate-intensity exercise (i.e., treadmill, swimming, and voluntary exercise) produced beneficial effects in young and adult GK rats. Studies seeking to determine the exercise intensity tolerance of GK rats still need to be performed.

Maximum VO_2_ (VO_2_ max) reflects an adaptation of the cardiorespiratory system and has been employed for validating clinical studies and calculating endurance work capacity (76). It has been reported that VO_2_ max rates between 50-70% correspond to moderate-intensity exercise (77,78). In this context, only two studies [Grijalva et al. (59) and Keller et al. (60)] mentioned the use of 50% VO_2_ max, which was based on a previous study (79). However, it is important to mention that the exercise intensity was not directly assessed in these studies, which can result in significant differences in relation to the markers evaluated in GK rats submitted to moderate physical exercise, as described in Supplementary Table S1.

Three studies used blood lactate analyses before and after exercise to quantify the exercise intensity (63,66,80). This analysis can detect the thresholds of sub-lactate (∼10 m/min, low-intensity) and supra-lactate (∼25 m/min, moderate-intensity). It is important to mention that sub-lactate threshold intensities have been suggested to promote no significant physiological changes associated with stress responses (81).

The main protocols that analyzed exercise intensities were defined by analyzing the VO_2_ max and lactate threshold. The primary intensities used were based on references from other rat strains with different diseases, consequently generating differences in metabolic responses in GK rats. As previously described, exercise intensity modulates several physiological parameters, including VO_2_ and cytochrome *c* levels (82). Moreover, regular physical activity or training at low to moderate intensity improves IR by increasing skeletal muscle fatty acid oxidation, augmenting hormonal responses (52,83) and modulating gene expression, protein signaling, and anti-inflammatory effects in GK rats (65). In this sense, extensive investigations evaluating the effect of exercise intensity on GK rat physiology and health are crucial.

As described in Supplementary Table S1 and Table 1, low to submaximal exercise intensity positively affects GK-T2DM metabolism. Positive effects are reported at intensities of 50-70% of the VO_2_ max, which corresponds to 15-21 m/min for one hour on the treadmill during each training session. The exercise programs occurred 3-5 times per week and ranged from 8 days to 15 weeks of training. However, it is important to note that exercise intensity varies depending on oxidative stress and other metabolic parameters (84).

**Table 1:** Exercise protocols used in each study and the physical intensity mentioned.

Article	Intensity (Low - Moderate)	Exercise protocol (Treadmill or swimming)
Tufescu et al. (2008) (48)	Not mentioned	20 m/min - 0% grade incline - 60 min/day
Grijalva et al. (2008) (59)	50% VO_2_ max	-
Kim S. et al. (2011) (52)	Not mentioned	21 m/min - 0% grade incline - 50 min/day
Qi et al. (2011) (49)	Not mentioned	20 m/min - 0% grade incline - 60 min/day
Kim M. et al. (2011) (87)	Not mentioned	120 min/swim - 15 min resting each 60 min/day
Salem et al. (2013) (62)	Not mentioned	20 m/min - 10% grade incline - 60 min/day
Keller et al. (2015) (60)	∼70% VO_2_ max (based on Soya et al. (81)	18 m/min until rat fatigue or 2 h
Raza et al. (2016) (28)	Not mentioned	20 m/min - 0% grade incline - 60 min/day
Macia et al. (2018) (61)	Not mentioned	20 m/min - 0% grade incline - 60 min/day
Morifuji et al. (2012) (66)	Sub-lactate, low intensity	15 m/min - 0% grade incline - 60 min/day
Tsutsumi et al. (2015) (80)	Sub-lactate, low intensity	15 m/min - 0% grade incline - 30 min/day
Kondo et al. (2015) (63)	Sub-lactate, low intensity	15 m/min - 60 min/day
Sinon et al. (2021) (88)	Moderate	15 m/min - 30 min/day

Moderate-intensity training was achieved when GK rats ran at 20 m/min (∼75% of the VO_2_ max), suggesting that this rat model, when trained, can perform moderate-intensity running for one hour (48). Notably, even low-intensity exercise protocols, such as walking or running at around 50% of the VO_2_ max until the submaximal effort is achieved enhance aerobic and anaerobic functions. This observation is due to exercise training-induced increases in eNOS activity, NO bioavailability, and subsequent vasodilation of the diabetic heart (59).

Brooks and White (85) reported differential effects in GK rats exposed to three conditions: a) rest; b) 14 m/min, at 1% inclination, easy exercise; and c) 28.7 m/min, at 15% inclination, heavy exercise. All exercise protocols lasted 90 minutes on a treadmill, where VO_2_, VCO_2_, and respiratory exchange ratio (RER = VCO_2_,/VO_2_) were determined to assess the metabolic responses. Interestingly, the classification and extrapolation of the proposed protocols (light, moderate, and heavy) did not directly mention the VO_2_ max intensity measurement.

Dudley et al. (86) used a training protocol with 15% inclination for 30, 60, and 90 min at speeds of 10, 20, 30, 40, 50, and 60 m/min. Exercise intensity-related differences were observed in the volumes of fast-twitch white (FTW), fast-twitch red (FTR), and slow-twitch red (STR) fibers and cytochrome *c* concentrations. For FTR, the best response was detected at 83% VO_2_ max (∼30 m/min) and between 50-75% VO_2_ max (10-20 m/min). The best response for STR was observed at 80% VO_2_ max (30-40 m/min). Moreover, exercise at VO_2_ max values of <50% did not generate significant mitochondrial adaptations and the responses between 60 and 90 min did not differ statistically.

One crucial point is that there are currently no studies utilizing the GK rat model in an incremental test, which would allow correlations to be made with the VO_2_ max in the same protocol. Most studies are based on Wistar, Wistar-Kyoto, Sprague-Dawley, and Okamoto-Aoki rat strains (76,82). It is also important to consider that, as analyzed by Bedford et al. (82), different VO_2_ max values vary among animal strain and gender, with male Sprague-Dawley exhibiting values of 85.2 mL·kg^-1^·min^-1^, female Sprague-Dawley, 81.1 mL·kg^-1^·min^-1^, male Wistar-Kyoto, 65.3 mL·kg^-1^·min^-1^, and male Okamoto-Aoki 72.3 mL·kg^-1^·min^-1^. Thus, studies assessing and evaluating the experimental approaches employed in exercise studies with the GK rat model are lacking.

Three articles mentioned using low-intensity exercise protocols, as determined by blood lactate levels (63,66,80). The protocol used by Tsutsumi et al. (80) demonstrated renoprotective action through the enhanced kidney-mediated AGE uptake. Additionally, Morifuji et al. (66) and Kondo et al. (63) reported that low-intensity exercise led to augmented SDH activity and stimulated pro-angiogenic factors [e.g., vascular endothelial growth factor (VEGF) and fetal liver kinase 1 (FLK-1)], consequently preventing microangiopathy and improving capillary communication. However, the protocols did not reduce blood glucose levels.

Macia et al. (61) described a reduction in plasma insulin levels promoted by low-intensity exercise. Along with this finding, increased citrate synthase activity, improved microcirculation, and up-regulated lactate and glucose metabolism-related protein expression (enzymes and transporters) were observed in the skeletal muscle.

Blood flow restriction combined with low-intensity electrical stimulation to induce muscle atrophy in GK rats prevented muscle mass loss, reduced AGE production and down-regulated the expression of receptor for AGE (RAGE), with no significant changes in blood glucose levels (72).

Using voluntary activity (i.e., low-intensity), Nakamoto et al. (65) and Kupai et al. (64) reported significant SDH activity in the soleus muscle and, unlike previous articles, lower blood glucose and HbA1c levels in exercised GK rats compared to rats not subjected to exercise. The total distance covered in the voluntary exercise could account for these discrepancies.

Kim et al. (87) used a swimming protocol to investigate the effects of physical exercise on GK rats but did not mention the exercise intensity level. The authors reported improved peripheral glucose utilization and insulin sensitivity. Tufescu et al. (48) demonstrated that exercise corresponding to 75% of the VO_2_ max (i.e., moderate-intensity) did not affect blood glucose levels but had a renoprotective effect that was enhanced by losartan.

Qi et al. (49) and Raza et al. (28) reported that moderate-intensity exercise contributes to resolving oxidative stress and improving mitochondrial function, as determined by cyclooxygenase 2 (COXII), glutathione reductase (GSH), p53 (a tumor-suppressor protein), TIGAR (p53 inducible gene), ATPase6, cytochrome B (CytB), and nuclear factor-kappaB (NF-κB) levels. These results suggested that moderate-intensity exercise improves energy metabolism and mitochondrial respiratory function, induces antioxidant responses, and restores insulin sensitivity.

Many of the processes involving eNOS are linked to mitochondrial biogenesis, which, in the case of diabetes, can lead to improved metabolic function in the face of moderate exercise intervention. Some of these points are related to basal mitochondrial activity maintenance, redox balance regulation, and vascular restoration, mediated by glucagon-like peptide-1 (GLP-1) through cAMP, which improves myocardial contractility via Akt. The eNOS activity is increased by enhancing the bioavailability of tetrahydrobiopterin (BH_4_), a cofactor for NO synthesis (59,60).

Of all the articles selected for this narrative review, around 27% mentioned the use of low or moderate intensity exercise, while the remaining studies (∼73%) did not mention the exercise intensity in the protocols used. Because direct markers of exercise training were not assessed in these studies, including VO_2_ max, lactate threshold, and enzyme activities, it is difficult to accurately define the exercise intensity and control loads used during training sessions in GK rat models (Table 1). Thus, we tried to address and discuss the main relevant results and differences from these previous studies, which is fundamental to guide and design further strategies to corroborate or refute the observed findings.

#### Beneficial effects of exercise in GK rats of different ages

**Figure 4:**
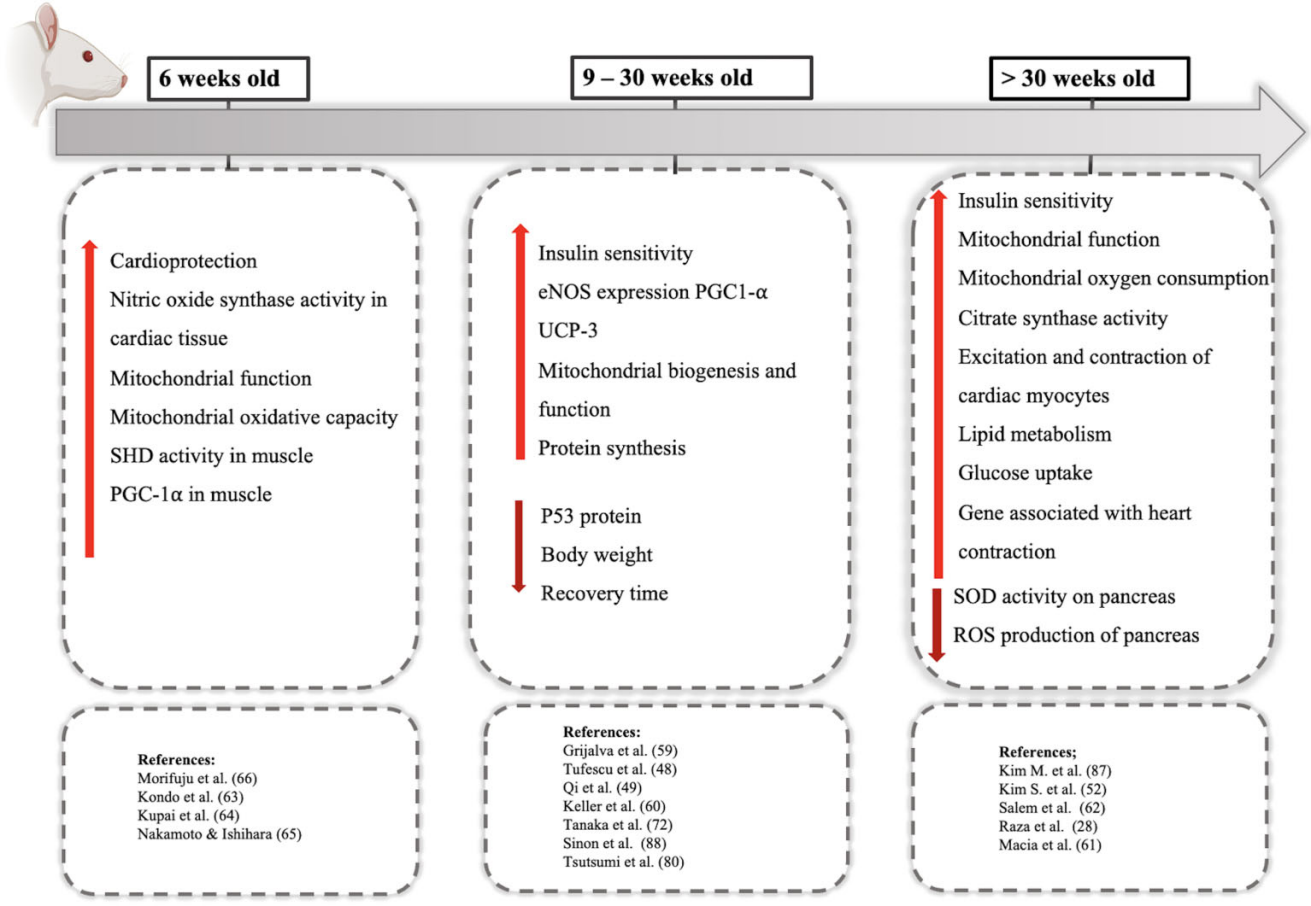
Summary of the physical exercise effects in Goto-Kakizaki (GK) rats of different ages.

A summary of the effects of physical exercise on GK rats of different ages is presented in Figure 4. It shows that a 3-6-week exercise program with young 6-12-week-old GK rats prevented microangiopathy, counteracting the capillary regression in skeletal and cardiac muscle and increased SDH and PGC-1α expression. Voluntary activity or low-intensity exercise attenuates infarct size by stimulating heme-oxygenase (HO) activity and effectively delaying the onset and progression of TDM2 in GK rats (63-66).

Similarly, training programs or physical muscle stimulation for 1, 2, 8, 9, 12, and 15 weeks at low- or moderate-intensity increased eNOS and PGC-1α expression and NO production, consequently eliciting positive kidney function effects. Exercise programs have also been shown to inhibit diabetes-associated muscle atrophy and enhance muscle protein synthesis in 6-30-week-old GK rats (48,49,59,60,72,80).

The effect of moderate-intensity exercise training for 4, 6, and 8 weeks was investigated in aged (>30 weeks of age) GK rats. The authors reported increased muscle and liver glycogen content, improved glucose homeostasis, oxidative metabolism, and IR, and up-regulated AMPK, PGC-1α, and GLUT-4 expression (28,52,61,62). The authors also observed improvements in ventricular myocyte Ca_2_^+^ handling despite variable muscle protein expression in GK rats. Exercise improved mitochondrial respiratory function and energy expenditure, induced an antioxidant response mechanism, reduced plasma insulin levels, and increased ATP production in exercising muscle. Glucose and lactate metabolism-regulating proteins were also up-regulated in the muscle and improved the recovery time of well-trained GK rats (28,52,61,62,87,88).

It is important to highlight that muscle function changes during life; the natural tendency during the aging process is the reduction in muscle protein balance, resulting in the reduction in muscle mass (89). Some strategies have been proposed to reduce or prevent muscle mass loss during aging, including nutritional and physical exercise programs, especially in people with comorbidities, such as obesity, type 2 diabetes mellitus, cardiovascular diseases, metabolic syndrome, among others, because these comorbidities exacerbate the process of muscle mass loss (3,65,67).

In this review, it was observed that physical activity and exercise training programs are potential strategies to generate important metabolic and physiological adaptations, leading to the improvement of several disturbances associated with type 2 diabetes mellitus in GK rats of all ages. Further studies are required to elucidate the mechanisms involved in these processes, as well as the long-term effect of physical exercise in preventing muscle mass loss during aging.

## Concluding Remarks

**Figure 5:**
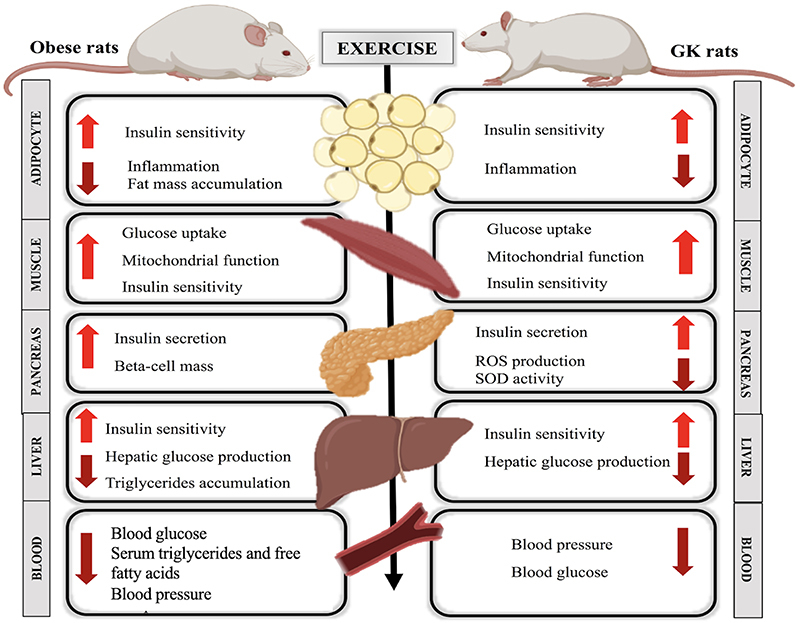
The effects of physical exercise on different tissues and organs of obese and non-obese (GK: Goto-Kakizaki) type 2 diabetes mellitus rats.

The exercise training protocols using GK rats cited in this review had beneficial effects on several T2DM features. Similar to obesity-related T2DM, these results indicated that moderate-intensity exercise training can attenuate or prevent complications in non-obese T2DM individuals. Furthermore, similar responses to exercise training were reported in the muscle, liver, adipose tissue, pancreas, and blood of obese and non-obese T2DM rats (Figure 5).

As discussed in this review, the diabetic characteristics and the protocols used to examine the regression of the T2DM condition confirmed that non-invasive treatments, such as physical exercise, promoted improvements in the physiometabolic response in GK and Wistar rats. However, it remains unclear if there is real health improvement or if physical exercise is an effective treatment. Further studies investigating how exercise intensity affects the responses and adaptation in non-obese T2DM animals are necessary. Filling in these knowledge gaps is essential for understanding the effects of exercise intensity (i.e., low, moderate, or high) and modality in different animal strains. Furthermore, cognitive and functional abilities need to be evaluated following exercise training (90). In conclusion, proper interventions focusing on a healthy lifestyle, including appropriate exercise protocols, are necessary to preserve the quality of life of both obese and non-obese T2DM patients.

## Supplementary Material

Click to view [pdf].
